# Balancing ethical data sharing and open science for reproducible research in biomedical data science

**DOI:** 10.1016/j.xcrm.2025.102080

**Published:** 2025-04-15

**Authors:** Dmitrijs Lvovs, Allison L. Creason, Stuart S. Levine, Michael Noble, Anup Mahurkar, Owen White, Elana J. Fertig

**Affiliations:** 1 Institute for Genome Sciences, University of Maryland School of Medicine, Baltimore, MD, USA; 2 Department of Medicine, Division of Hematology/Oncology, University of Maryland School of Medicine, Baltimore, MD, USA; 3 Knight Cancer Institute, Oregon Health Science University, Portland, OR, USA; 4 Biomedical Engineering Department, Oregon Health & Science University, Portland, OR, USA; 5 BioMicro Center, Department of Biology, Massachusetts Institute of Technology, Cambridge, MA, USA; 6 Break Through Cancer, Cambridge, MA, USA; 7 University of Maryland - Institute for Health Computing, Bethesda, MD, USA; 8 Greenebaum Comprehensive Cancer Center, University of Maryland School of Medicine, Baltimore, MD, USA

## Abstract

Analyses of large-scale health data in biomedical data science can help uncover new treatments and deepen our understanding of disease and fundamental biology. Here we examine the balance between ethical and responsible data sharing and open science practices that are essential for reproducible research in biomedical data science.

## Main text

### Introduction

**Figure 1:**
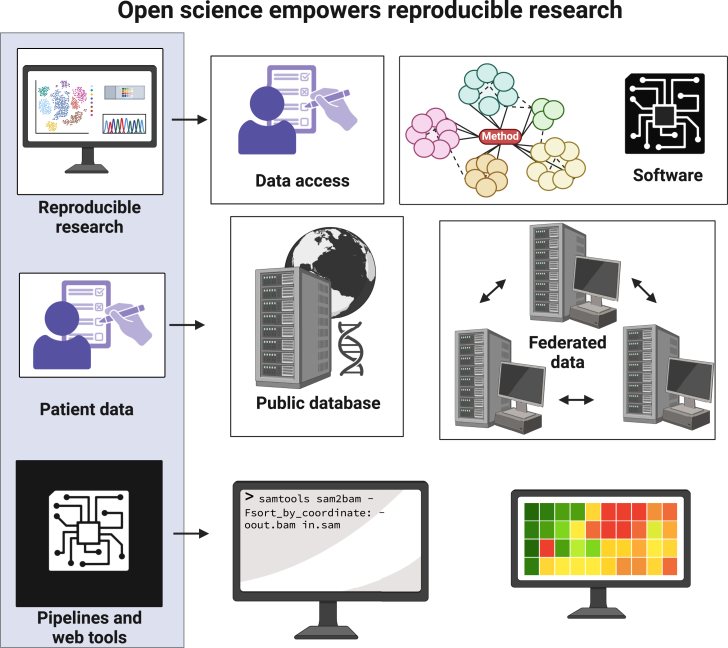
Open science empowers reproducible research Reproducible research requires open data and methods implemented as open-source software. Secured data platforms help ensure balance between the privacy of sensitive data and reproducibility best practices. Workflow engines control the execution of a series of methods to achieve a complex analytical goal.

Recent advances in artificial intelligence (AI), digitized clinical data, and high-throughput molecular and cellular measurement technologies have brought data science to the forefront of biomedical research. Leveraging large-scale multidimensional health data can improve early diagnoses, discover new treatments, and aid our understanding of disease. The translation of large-scale data (e.g., generated using omics technologies) into clinical decisions requires data analysis to be directly focused on improving patient outcomes, enhanced for clinical practice with interpretable machine learning methods. Data science, a growing -discipline that uses computer programming, statistical analysis, and machine learning to generate knowledge, has advanced open-source software and improved access to data for data-driven biomedical tools in reproducible research (Figure 1). The transparency these practices enable must be balanced with the security of patient data and clinical action due to the impact these discoveries can have on patient health care.

### Open science and data sharing promote transparency and reproducibility in biomedical data science

Lessons learned from failures in data-driven biomedical research in the early days of microarrays led to the adoption of open science and reproducible research as best practices for data-driven biomedical research.^1^ Because data science is computationally driven, all results should be automatically reproducible from the same dataset if the analysis code is readily available. This model of reproducible research enables investigators to verify that the findings are accurate, reduce biases, promote scientific integrity, and build trust. Open science, the movement that promotes very broad dissemination of research associated resources in general, and FAIR^2^ (findable, accessible, interoperable, reusable), the best-practice guidelines for data stewardship in research, promote data and software that are widely shared, well-described, consistent, and reproducible.

Transparency in research involves openly sharing methodologies, data, and findings with the community. Best practices for reproducible research have been well established and require complete data and code sharing.^3^ Open science also allows the scientific community to build upon previous work to expand the reach of biomedical data science across disease systems and therapeutic modalities. Despite its importance to the research community due to providing additional funding mechanisms and application focus, the incentives to commercialize biomedical research can sometime place barriers on the complete code and data sharing transparent research requires.

### Ethical considerations in data sharing to protect human privacy

The requirement of open data for reproducible research must be balanced with adequate actions to ensure privacy, including implementing measures to protect sensitive information from unauthorized access or breaches. Legal and regulatory requirements vary by region and must be followed to ensure compliance with data protection laws. In the United States, the National Institutes of Health (NIH) set out expectations of broad and responsible sharing of genomic data generated from its funded research (accessible at https://grants.nih.gov/grants/guide/notice-files/not-od-14-124.html). NIH introduced a standardized process for sharing sensitive data that involves collaborating principal investigators and institutional review boards (IRBs) of the releasing and receiving institutions, providing a foundation for ethical genomic data sharing. IRBs play a vital role in determining data release, storage, and access requirements in the institutions internally.

The value of either clinical or research-derived genomic data achieves maximal scientific utility when well-characterized phenotypic data are available and accessible. Phenotypic data includes information about an individual’s physical characteristics, health status, and medical history and is essential for understanding the relationship between molecular variations and health outcomes. Electronic health records contain protected health information (PHI) that is safeguarded by national legislations such as the Health Insurance Portability and Accountability Act (HIPAA) in the US and the General Data Protection Regulation (GDPR) in the EU.

While raw genomic data and PHI are directly identifiable and are well guarded, other important clinically relevant factors such as age, environment, or some gene variants pose a risk of re-identification and raise ethical considerations for fully open science. Understanding which phenotypic and derived genotypic data are potentially identifiable is crucial, especially in vulnerable or small populations. Thus, proper classification of both raw and summary data would ensure ethical and compliant sharing. Institutions with a strong understanding of data classification^4^ develop tiers based on the risk of re-identification to govern their internal data management practices.

Ethical data sharing involves obtaining explicit informed consent from participants. Informed consent means that participants are fully aware of how their data will be used, shared, and protected; consent should be captured and accompany the data records for ethical use.

Federated data systems that bring analysis software to clinical datasets across institutions have been shown to enable biomedical data science and reproducible research without having to replicate data on multiple systems.^5^ Behind the scenes, such systems either import all the searchable metadata into a centralized location (an approach taken by the Common Fund Data Ecosystem^6^) or distribute the search through federated searches, as implemented in the Global Alliance for Genomic Health (GA4GH).^7^

### Ensuring data quality and mitigating batch effects by tracking metadata

Not all data streams hold the same value in biomedical data science. The quality of data is just as crucial for clinical translation as the amount of data. Systematic data collection, quality assessment (tailored for different data modalities), normalization, and artifact removal ensure that analysis reflects true biological signals.

Interpreting biomedical data into clinical and biological knowledge requires the application of data analysis or machine learning methods. Before these methods can be applied, each source of data should go through a series of quality checks and preprocessing for standardization. Quality assessment involves evaluating data for errors, inconsistencies, and missing values. Normalization involves adjusting data to account for variations in measurement techniques or experimental conditions. In many cases, these technical errors can be mitigated by systematic data collection with standardized protocols and processes designed to collect data consistently.

Despite best practices in data collection, unavoidable technical artifacts like batch effects pervade almost all high-throughput data.^8^ Such artifacts can obscure true biological signals and lead to incorrect conclusions. Artifact removal involves identifying and eliminating sources of noise or bias in the data. Several batch-effect correction algorithms have been developed that use computational approaches to mitigate technical artifacts while preserving biological variation. Selecting appropriate batch-effect correction methods requires understanding the dataset, technical factors, assumptions, and analysis goals. But no algorithm can remove technical artifacts that are perfectly confounded with the clinically relevant findings, so study designs that foster balance between technical groups and outcomes are essential to analysis.^9^ This process can be enhanced by combining data across distinct medical centers, making it essential to consider both hospital performance and disparities in patient populations when designing and analyzing sample cohorts.

The plausible way to assess the impact of technical artifacts on data quality is to have access to all the information related to the data generation. Metadata is information that describes the data, including details about how it was collected, processed, and analyzed. Accurate metadata that encodes these technical artifacts is key to this tracking, providing the basis for FAIR principles. Capturing and tracking metadata during data generation is crucial, ensuring that important details are not lost or forgotten. The creation of community standards for metadata ensures that the most pertinent variables are captured for analysis accuracy. It should be noted that making metadata standards too extensive can pose a considerable practical barrier to researchers recording them. These limitations can be overcome with data management platforms that gather comprehensive metadata more automatically for researchers, while simultaneously improving the reproducibility and reuse of datasets by providing a clear record of the data’s provenance.^10^

### Data harmonization is supported through community standards and data sharing resources

The heterogeneity of metadata and data streams in multi-modal data challenges data sharing. Data harmonization and standardization efforts enhance integration and are supported by data sharing platforms that require specific data formatting guidelines to be met and minimum acceptable metadata to be present for the data that is shared. Meeting such requirements requires significant resources that are not tied directly to the scientific goals of most projects. The adoption of common standards, ontologies, and innovative automated systems is necessary to address these challenges.

Data harmonization involves aligning data from different sources to ensure consistency and compatibility. Standardization involves adopting common formats, terminologies, and protocols for data collection and reporting. Biomedical research communities often define ontologies to categorize and encode these terminologies into standardized language to facilitate harmonization and standardization across studies. These efforts enhance the integration of data across resources, enabling researchers to combine and compare datasets and support collaborative cross-institutional research.^11^ Data harmonization and standardization require significant investment of time, resources, and expertise. Innovative automated systems, including advances to AI for these tasks, can help streamline these processes and improve efficiency.^12^

In addition to standardization, the biomedical research community also needs tools to access standardized and curated datasets for data-science research. A wealth of biomedical data-sharing resources exists, including genomic and multi-omics repositories, clinical and phenotypic data repositories, public health platforms, drug discovery repositories, and general open science platforms (a comprehensive list is available at https://www.nlm.nih.gov/NIHbmic/bmic-about.html). These resources provide structured access to vast amounts of trusted biomedical data, accelerating discovery and improving human health.

Often, the specific data-sharing platforms, file formats, and ontologies vary for each data stream in biomedical data science. Genomic and multi-omics repositories store data related to genetic and molecular studies. Clinical and phenotypic data repositories store information about patient characteristics, health status, and medical history. Public health platforms store data related to disease surveillance, health outcomes, and population health. Drug discovery repositories store data related to drug development and bioactivity. The combined data size and rapid advancement to imaging and spatial molecular platforms have made the development of similar platforms for these data modalities an area of active research.

The data type specificity of many biomedical data-sharing platforms poses a challenge to analyses that span multiple data streams. While challenging, facilitating linkage between data portals and data types will empower the multi-modal data analysis that is essential to comprehensively map the systems-level determinants of disease and therapeutic response. However, it is important to note that applying analyses from these multimodal datasets can make new and unanticipated discoveries about a patient’s health. This poses ethical considerations, in which a clinician may have an obligation to notify a patient about this observation. This potential must be considered in the initial patient consent that allows for input of these data into these platforms and linkage between platforms. Moreover, data input must be limited and the analysis tools in the data platforms themselves must be limited to ensure that novel findings made from their input data do not extend beyond the conditions to which a patient consented for use of their data.

### Open source analysis tools and software platforms aid reproducible research

Computational analysis methods are needed to interpret digitized biomedical data streams into clinical and biological insights. Methods are encoded into software tools that enable the analysis. Biomedical data science often requires a series of multiple tools, referred to as analysis pipelines. Just as an experimental protocol would document all the details of each experiment in the methods, so too should biomedical data scientists document carefully each of the software tools, parameters, and versions used for analysis for fully reproducible research. The computer code used for analysis serves as the reference for the analysis approach. In principle, this code can provide both documentation and the ability to fully reproduce analysis results. Tools such as Sweave, knitr, and Jupyter notebooks have emerged to embed results with the code to enable fully reproducible research that can be shared as paper supplements. Git enables tracking of changes to this analysis code, and platforms such as GitHub, GitLab, and Bitbucket promote collaboration and code sharing. Resources including CodeOcean and Zenodo allow the capture of the exact version of code in a way suitable for publication in journals. The biomedical data science research community nearly unanimously endorsed fully releasing the software code, a practice that is referred to as open source.

The fundamental computational methods and software development for their implementation are an active area of research in biomedical data science. Although often required to be customized to projects, computational researchers strive to develop methods and software that can be reused consistently in different research applications. Reproducibility and ease of use are not a substitute for the accuracy of the underlying method. A further advantage of open source is that the entire research community can assess the accuracy of the approaches via benchmarking, help identify errors, and propose improvements for the most accurate results. Benchmarking methods using synthetic or open data can provide good initial insights into method applicability, but study designs with robust test and training sets from independent cohorts are important for model validation. It is important that these assessments be performed on cohorts of patients from all backgrounds and clinical settings to mitigate bias in performance and promote equity in access to computational biomedical tools.

Community ecosystems, including notably Bioconductor and Biopython, have developed to provide standards for the development of quality biomedical data science tools. While featuring speed, the community-wide development of biomedical data science software often leads to frequent updates and changes that can hinder reproducibility. Container images, such as Docker, isolate packages that fully capture the software version and computing environment to ensure reproducible analyses while software updates continue in the wild. In all cases, maintaining software can be challenging for research labs that rely on innovation and publications for promotion standards and lack consistent funding streams to fund software developers for their sustainment.^13^ Biomedical data science uses a series of analysis methods to achieve a complex analysis goal. Combining tools into reproducible workflows is essential for such complex analyses. Workflow engines such as Nextflow^14^ enable reproducible workflows by combining multiple tools and ensuring their joint execution across the heterogeneous computational resource landscape. These often require researchers to work at the command line or on computer servers, which can have a steep learning curve for researchers not trained in computer coding.

User-friendly tools make it easier for researchers to analyze and visualize data, even if they lack advanced computational skills. Further, interactive visualization and human-in-the-loop analysis are often vital for interpreting complex datasets. Web-based data visualization resources, like cBioPortal and Xena Browser, enable interactive exploration of large-scale genomics datasets. The point-and-click nature of these tools allow for easy implementation of multiple analysis steps, but reproducibility can be lost if a researcher does not track the details of each analysis step they selected. Software that maintains a log of each step that users have taken can help support reproducibility and user-friendliness for web-based exploratory analysis tools. The extensive coding and visualization work required for developing these web-based analysis applications can limit the ability of their developers to adapt to the rapid updates to biomedical software from the open-source research community. Workflow platforms like Galaxy, Seqera, and GenePattern Notebook serve as an intermediary that enable researchers to perform complex analyses that are fully tracked and rely on best-practice open-science tools without themselves needing to write code. These software approaches have traditionally relied on the input of data streams, which can pose additional security risks of sharing patient data to perform an analysis. Cloud platforms like AnVIL provide both a secure environment for data sharing and analysis, and a distributed data system to enhance data accessibility. These user-friendly platforms that bring computational methods to the data ensure data privacy and security. As a result, they can enable the balance of best practices in accessible, reproducible research while simultaneously securing patient data and allowing for ready adaptation to federated biomedical data.

### Conclusion

Continued improvements in data-sharing infrastructure, analysis software, and policies enforcing open and reproducible research are necessary to address challenges and maximize the benefits of shared data. Coupling data sharing with open-source analysis tools is fundamental to advancing biomedical research; it promotes transparency for clinical decision making and facilitates collaboration and clinical translation from biomedical data. Ethical and responsible data sharing democratizes research, supports the advance of AI, and informs public health policies. By addressing these challenges and promoting ethical and responsible data sharing, the biomedical research community can maximize the benefits of shared data, accelerate discovery, and improve human health.

## Acknowledgments

The authors were funded by the Break Through Cancer Data Science TeamLab (D.L., S.S.L., M.N., E.J.F.); NIH Common Fund OT3OD025459-01 (A.M., O.W.); NIH U01CA294548 (A.L.C., E.J.F.); U24CA284156 (E.J.F.); P30CA134274 (D.L., E.J.F.); U54AG079779 (E.J.F.); P30CA14051P42 (S.S.L.); P42ES027707 (S.S.L.); and the UM-IHC MPower (E.J.F. and A.M.).

## Declaration of interests

E.J.F. was on the scientific advisory board of Resistance Bio/Viosera Therapeutics and a paid consultant for Mestag Therapeutics and received research grants from Abbvie Inc and Roche/Genetech outside the scope of this commentary.

## Declaration of generative AI and AI-assisted technologies in the writing process

During the preparation of this work the author(s) used Bing Copilot to summarize inputs from multiple authors and to split the text into logical paragraphs. After using this tool/service, the author(s) reviewed and edited the content as needed and take(s) full responsibility for the content of the published article.
